# Evaluation of sulphonamide derivatives acting as inhibitors of human carbonic anhydrase isoforms I, II and *Mycobacterium tuberculosis***β**-class enzyme Rv3273

**DOI:** 10.1080/14756366.2018.1471475

**Published:** 2018-05-18

**Authors:** Tanvi V. Wani, Silvia Bua, Pravin S. Khude, Abdul H. Chowdhary, Claudiu T. Supuran, Mrunmayee P. Toraskar

**Affiliations:** a Department of Pharmaceutical Chemistry, Bharati Vidyapeeth’s College of Pharmacy, Navi Mumbai, India;; b NEUROFARBA Department, Sezione di Scienze Farmaceutiche, Università degli Studi di Firenze, Sesto Fiorentino, Florence, Italy

**Keywords:** Carbonic anhydrase, sulphonamide, human isoforms I and II, *Mycobacterium tuberculosis*

## Abstract

A series of novel sulphonamide derivatives was obtained from sulphanilamide which was N4-alkylated with ethyl bromoacetate followed by reaction with hydrazine hydrate. The hydrazide obtained was further reacted with various aromatic aldehydes. The novel sulphonamides were characterised by infrared, mass spectrometry, ^1^H- and ^13^C-NMR and purity was determined by high-performance liquid chromatography (HPLC). Human (h) carbonic anhydrase (CA, EC 4.2.1.1) isoforms hCA I and II and *Mycobacterium tuberculosis* β-CA encoded by the gene Rv3273 (mtCA 3) inhibition activity was investigated with the synthesised compounds which showed promising inhibition. The K_I_s were in the range of 54.6 nM–1.8 µM against hCA I, in the range of 32.1 nM–5.5 µM against hCA II and of 127 nM–2.12 µM against mtCA 3.

## 1. Introduction

Sulphonamides are interesting biologically active compounds. There are numerous sulphonamide drugs available on the markets for the treatment of various diseases^1^. Sulphonamide derivatives such as acetazolamide, methazolamide, ethoxzolamide, dichlorophenamide, dorzolamide and brinzolamide have been clinically used for decades as inhibitors of the zinc enzyme carbonic anhydrase (CA, EC 4.2.1.1). A diverse research trend in the past years has led to the obtaining of diuretic, anti-glaucoma, anti-cancer, anti-convulsant, anti-diabetic, and anti-obesity agents based on CA inhibitors (CAIs) of the sulphonamide type^2–6^. Sulphonamides act as strong CAIs by binding as anions to the zinc metal ion within the enzyme active site^7^.

CA has various roles in physiological events such as carbon dioxide and bicarbonate transport processes, respiration, pH balancing, CO_2_ homeostasis, electrolyte secretion, biosynthetic reactions^1^. Distinct, evolutionarily non-related gene families of CAs are present in various organisms, out of which the α-class is present in humans, as 15 different isoforms (hCA I–XIV). hCA I is present in red blood cells and in many tissues but its physiological function is still unknown; however, it is known that hCA I is associated with retinal and cerebral edema, and the inhibition of CA I may be helpful in curing such conditions^1–5^. hCA II, the physiologically dominant isoform, is another enzyme which is associated with several disease conditions such as epilepsy, edema, glaucoma and altitude sickness^1–5^. Furthermore, it has also emerged in the past few years that these enzymes can be used as potential target for designing anti-infective drugs with a novel mechanism of action^6^.

In this article, we report a synthetic strategy for the generation and characterisation of some sulphonamide derivatives. The novel sulphonamides were purified and characterised using IR, Mass spectrometry, ^1^H and ^13^C-NMR for confirmation of their structure, and purity of the compounds was determined by using HPLC techniques. The newly synthesised compounds were analysed as inhibitors of human hCA I and II, and the bacterial, β-class enzyme from *Mycobacterium tuberculosis* (mtCA 3) encoded by the gene Rv3273^8^.

## 2. Material and method

### 2.1. Chemistry

All the reagents and solvents were obtained from commercial suppliers and were used as received unless otherwise indicated. Solvents were dried, wherever necessary, according to standard procedures. All reactions were performed under N_2_ atmosphere, unless otherwise indicated. Analytical silica gel 60 F_254_-coated TLC plates were purchased from Sigma-Aldrich (Milan, Italy), and were visualised with UV light. IR spectra (ATR) were recorded on a Quest ATR Diamond Accessory (Black) P31482 & Shimadzu 8100 infrared spectrophotometer. ^1^H-NMR was recorded at 300 MHz in DMSO-d_6_ as solvent using TMS as an internal reference standard at Sophisticated Analytical Instrument Facility (SAIF). Molecular ion peaks of some of the synthesised compounds were recorded using LCMS at Laxai- Avanti Life Sciences Pvt. Ltd Hyderabad, India. Melting points were recorded using a Veego^®^ (VMP)-D capillary melting point apparatus (Veego Instruments Corp., Mumbai, India) and are uncorrected. Percent Purity of synthesised compounds was determined by performing RP-HPLC.

#### General procedure for synthesis of ethyl 2-((4-sulphamoylphenyl) amino) acetate (2)^9^

In a solution of sulphanilamide (0.01 mol) in absolute ethanol (20 ml), ethyl bromoacetate (0.01 mol) and anhydrous potassium carbonate (0.6 g) were added and the reaction mixture was heated under reflux for 12 h. The potassium salt was filtered off and excess of ethanol was removed under reduced pressure. The residue solidifies on cooling to give compound (**2**). Yield: 70%, R_f_: 0.72 (chloroform:methanol 9:1); M.P.: 141–144 °C; IR (ATR) cm^−1^: 3356 (N–H *str* of NH_2_), 3271 (N–H *str*), 2993 (Ar C–H *str*), 2904 (aliphatic C–H *str*), 1728 (C = O *str*), 1597 (C = C *str*), and 1138 (S = O *str*).

#### General procedure for synthesis of 4-((2-hydrazinyl-2-oxoethyl)amino) benzenesulphonamide (3)^10^

In a solution of compound (**2**) (0.01 mol) in absolute ethanol (15 ml), hydrazine hydrate (0.02 mol) was added and the reaction mixture was heated under reflux for 4 h. The excess of ethanol was removed under vacuum and the reaction mixture was allowed to cool. The reaction mixture was then diluted with ice-cold water. The precipitate obtained was filtered, washed with cold water, dried and recrystallised from ethanol. Yield: 50%; R_f_: 0.46 (chloroform:methanol 9:1); M.P.: 174–177 °C, ^1^H-NMR (300 MHz, DMSO-d_6_) δ ppm: 9.15 (s, 1H, −CONH), 7.51–7.47 (dd, 2H, Ar–H), 6.93 (s, 2H, −SO_2_−NH_2_), 6.62–6.61 (dd, 2H, Ar**–**H), 6.58 (t, 1H, Ar–NH), 4.25 (d, 2H, −CH_2_−), 3.69–3.67 (d, 2H, −NH_2_); IR (ATR, cm^−1^): 3344 (N − H *str* of NH_2_), 3294 (N − H *str*), 3034 (Ar C − H *str*), 2943 (aliphatic C**–**H *str*), 1654 (C = O *str*), 1589 (Ar C = C), and 1139 (S = O *str*).

#### General procedure for synthesis of (E)-1-(4-chlorophenyl)-3-phenylprop-2-en-1-one derivatives (4a–4k)^11–13^

4-Chloro-acetophenone (0.01 mol) and various substituted aromatic aldehydes (0.01 mol) were mixed in ethanol (40 ml) in a conical flask placed in an ice bath. To this, 60% NaOH solution (10 ml) was added dropwise with continuous stirring for 30 min. The mixing was continued for another 2–3 h maintaining the ice bath. The mixture was kept in a refrigerator overnight. Reaction completion was confirmed by TLC (hexane:ethyl acetate = 2:1). Then, it was diluted with ice-cold water, filtered, washed well with cold water, dried in air and recrystallised from rectified methanol.

#### General procedure for synthesis of 4-aryl/heteroaryl but-3-en-2-one derivatives (4l–4u)^14^

A mixture of aldehyde (1 equiv) and acetone (13.6 equiv) was added in aqueous 60% NaOH solution. The mixture was stirred at 40 °C. Reaction completion was confirmed by TLC (hexane:ethyl acetate = 2:1). On completion of reaction, 25 cm^3^ of water were added to reaction mixture to afford the crude as an oil. The product was extracted with AcOEt (3 × 25 cm^3^) and dried in air. Reaction with 4-((2-hydrazinyl-2-oxoethyl)amino) benzenesulphonamide (**3**) led to the final products.

#### General procedure for synthesis of 4-((2-(3-(4-chlorophenyl)-5-aryl/heteroaryl-4,5-dihydro-1H-pyrazol-1-yl)-2-oxoethyl)amino)benzenesulphonamide derivatives (5a–5k)^15^

A mixture of chalcone (*E*)-1-(4-chlorophenyl)-3-*aryl/heteroaryl*-prop-2-*en*-1-one derivatives (4a–4k) (0.01 mol) and 4-((2-hydrazinyl-2-oxoethyl) amino) benzenesulphonamide (**3**) (0.02 mol) in 20 ml ethanol was refluxed for 2 h. To this, alcoholic KOH solution (10 ml, 60%) was added dropwise with continuous stirring for 30 min. The reaction mixture was refluxed further for 2 h and stirred overnight. Reaction was monitored by TLC using chloroform:methanol (0.8:0.2). The resulting solution was poured on ice-cold water. Precipitate obtained was filtered and recrystallised from ethanol.

#### General procedure for synthesis of 4-((2-(3-methyl-5-ary/heteroaryl-4,5-dihydro-1H-pyrazol-1-yl)-2-oxoethyl)amino)benzenesulphonamidederivatives (5l–5u)^16^

4-Aryl/heteroaryl-but-3-*en*-2-one derivatives (**4l–4u)** (0.01 mol) were dissolved in hot glacial acetic acid. To this solution 4-((2-hydrazinyl-2-oxoethyl)amino)benzenesulphonamide (**3**) (0.015 mol) was added and refluxed. Reaction was monitored by TLC using chloroform:methanol (9:1). The resulting solution was poured on ice-cold water. Precipitate obtained was filtered and recrystallised from 90% ethanol.

#### General synthesis of 4-((2-(arylmethylidene)hydrazinyl)-2-oxoethyl)amino)benzene sulphonamide derivatives (6a–6j)^17–19^

In a 250-ml flask, equimolar quantity of hydrazide **(3)** (0.05 mol) and aromatic aldehyde (0.05 mol) were dissolved in EtOH (50 ml). Glacial acetic acid (2–3 ml) was added to adjust the pH to 5–6. The reaction mixture was refluxed for 3 h. The progress of reactions was monitored using TLC using hexane:ethyl acetate (8:2) as the mobile phase. After completion of reaction (as seen from TLC), the mixture was poured onto the crushed ice and the precipitated product was filtered, washed twice with ice-cold water, dried and recrystallised from water:ethanol.

Using the above general procedure, the following compounds were prepared and characterised.

#### 4-((2-(3-(4-Chlorophenyl)-5-phenyl-4,5-dihydro-1H-pyrazol-1-yl)-2-oxoethyl)amino) benzenesulphonamide (5a)

Yield: 70%; R_f_: 0.53 (chloroform:methanol 9:1); M.P.: 140–144 °C; purity (HPLC): 88.87%; ^1^H-NMR (300 MHz, DMSO-d_6_) δ ppm: 7.98–7.86 (dd, 2H, Ar–H), 7.57–7.50 (m, 4H, Ar–H), 7.47–7.20 (m, 5H, Ar–H), 6.93 (s, 2H, –SO_2_NH_2_), 6.67–6.64 (dd, 2H, Ar–H), 6.59–6.55 (t, 1H, Ar–NH), 5.61–5.56 (dd, 1H, Hx), 4.49–4.30 (dd, 2H, –CH_2_–), 3.93–3.83 (dd, 1H, Ha), 3.21–3.13 (dd, 1H, Hb); ^13^C-NMR (100 MHz, DMSO-d_6_): δ = 167.07, 154.42, 151.38, 142.22, 135.8, 131.15, 130.11, 129.17, 129.00, 128.85, 127.67, 125.91, 111.62, 60.61, 45.15, 42.2; IR (ATR, cm^−1^): 3390, 3306 (N–H *str* of NH_2_), 3234 (N–H *str*), 3074 (Ar C–H *str*), 2939 (aliphatic C–H *str*), 1683 (C = O *str*), 1155 (S = O *str*), 738 (C–Cl *str*); LC-MS (ESI; M)^+^: 468.5.

#### 4-((2-(3-(4-Chlorophenyl)-5-(4-fluorophenyl)-4,5-dihydro-1H-pyrazol-1-yl)-2-oxoethyl)amino) benzenesulphonamide (5b)

Yield: 68%; R_f_: 0.46 (chloroform:methanol 9:1); M.P.: 179–181 °C; purity (HPLC): 98.02%; ^1^H-NMR (300 MHz, DMSO-d_6_) δ ppm: 7.86–7.84 (dd, 2H, Ar–H), 7.54–7.49 (m, 4H, Ar–H), 7.28–7.24 (dd, 2H, Ar–H), 7.10–7.06 (t, 2H, Ar–H), 6.89 (s 2H, −SO_2_NH_2_−), 6.66–6.64 (dd, 2H, Ar–H), 6.48–6.45 (t, 1H, Ar–NH), 5.62–5.58 (dd, 1H, Hx), 4.46–4.30 (dd, 2H, −CH_2_−), 3.91–3.84 (dd, 1H, Ha), 3.20–3.15 (dd, 1H, Hb); ^13^C-NMR (100 MHz, DMSO-d_6_): δ = 167.19, 160.64, 154.47, 151.43, 138.36, 135.78, 131.17, 130.12, 129.22, 128.94, 128.14, 127.67, 115.85, 111.65, 59.98, 45.15, 42.12; IR (ATR, cm^−1^): 3390, 3308 (N–H of NH_2_), 3236 (N–H *str*), 3074 (Ar C–H *str*), 2924 (aliphatic C–H *str*), 1683 (C = O *str*), 1157 (S = O *str*), 738 (C–Cl *str*); LC-MS (ESI; M)^+^: 486.8.

#### 4-((2-(3-(4-Chlorophenyl)-5-(4-bromophenyl)-4,5-dihydro-1H-pyrazol-1-yl)-2-oxoethyl)amino) benzenesulphonamide (5c)

Yield: 75%; R_f_: 0.44 (chloroform:methanol 9:1); M.P.: 182–185 °C; purity (HPLC): 99.11%; ^1^H-NMR (300 MHz, DMSO-d_6_) δ ppm: 7.88–7.86 (dd, 2H, Ar–H), 7.57–7.47 (m, 4H, Ar–H), 7.29–7.20 (dd, 2H, Ar–H), 7.16–7.10 (t, 2H, Ar–H), 6.93 (s, 2H, –SO_2_NH_2_–), 6.66–6.63 (dd, 2H, Ar–H), 6.58–6.55 (t, 1H, Ar–NH), 5.62–5.56 (dd, 1H, Hx), 4.42–4.35 (dd, 2H, –CH_2_–), 3.92–3.82 (dd, 1H, Ha), 3.22–3.15 (dd, 1H, Ha); ^13^C-NMR (100 MHz, DMSO-d_6_): δ = 166.81, 151.5, 142.96, 137.27, 133.84, 131.56, 131.09, 129.27, 128.74, 128.19, 127.7, 123.78, 111.62, 44.00; IR (ATR, cm^−1^): 3362, 3300 (N–H of NH_2_), 3230 (N–H *str*), 3080 (Ar C–H *str*), 2846 (aliphatic C–H *str*), 1683 (C = O *str*), 1155 (S = O *str*), 736 (C–Cl *str*), 580 (C–Br *str*).

#### 4-((2-(3-(4-Chlorophenyl)-5-(4-methylphenyl)-4,5-dihydro-1H-pyrazol-1-yl)-2-oxoethyl) amino)benzenesulphonamide (5d)

Yield: 70%; R_f_: 0.57 (chloroform:methanol 9:1); M.P.: 151–154 °C; purity (HPLC): 100%; ^1^H-NMR (300 MHz, DMSO-d_6_) δ ppm: 7.85–7.80 (dd, 2H, Ar–H), 7.58–7.50 (m, 4H, Ar–H), 7.10 (s, 4H, Ar–H), 6.89 (s, 2H, SO_2_NH_2_), 6.70–6.64 (dd, 2H, Ar–H), 6.45–6.42 (t, 1H, Ar–NH), 5.57–5.53 (dd, 1H, Hx), 4.46–4.30 (dd, 2H, CH_2_), 3.98–3.81 (dd, 1H, Ha), 3.18–3.12 (dd, 1H, Hb), 2.27 (s, 3H, Ar–CH3); ^13^C-NMR (100 MHz, DMSO-d_6_): δ = 167.02, 154.44, 151.4, 139.28, 137.23, 135.76, 131.16, 130.32, 129.19, 128.84, 128.14, 127.69, 125.89, 111.64, 60.41, 45.16, 42.2, 21.12; IR (ATR, cm^−1^): 3362, 3294 (N–H of NH_2_), 3232 (N–H *str*), 3082 (Ar C–H *str*), 2853 (aliphatic C–H *str*), 1680 (C = O *str*), 1151 (S = O *str*), 740 (C–Cl *str*).

#### 4-((2-(3-(4-Chlorophenyl)-5-(pyridin-4-yl)-4,5-dihydro-1H-pyrazol-1-yl)-2-oxoethyl)amino) benzenesulphonamide (5e)

Yield: 71%; R_f_: 0.41 (chloroform:methanol 9:1); M.P.: 161–164 °C; ^1^H-NMR (300 MHz, DMSO-d_6_) δ ppm: 8.5 (s, 2H, Pyr–H), 7.88–7.85 (dd, 2H, Ar–H), 7.57–7.51 (m, 4H, Ar–H), 7.26–7.24 (dd, 2H, Ar–H), 6.93 (s, 2H, SO_2_NH_2_), 6.68–6.58 (dd, 2H, Ar–H), 6.61–6.58 (t, 1H, Ar–NH), 5.63–5.57 (dd, 1H, Hx), 4.54–4.32 (dd, 2H, CH_2_), 3.95–3.85 (dd, 1H, Ha), 3.25–3.17 (dd, 1H, Hb); ^13^C-NMR (100 MHz, DMSO-d_6_): δ = 168.78, 154.19, 151.09, 149.92, 149.12, 135.22, 130.73, 129.75, 128.86, 127.18, 120.81, 111.24, 59.23, 44.56, 41.12; IR (ATR, cm^−1^): 3390, 3309 (N–H of NH_2_), 3238 (N–H *str*), 3074 (Ar C–H *str*), 2926 (aliphatic C–H *str*), 1685 (C = O *str*), 1521 (C = N *str*), 1153 (S = O *str*), 736 (C–Cl *str*); LC-MS (ESI; M)^+^: 469.10.

#### 4-((2-(3-(4-Chlorophenyl)-5-(2-chlorophenyl)-4,5-dihydro-1H-pyrazol-1-yl)-2-oxoethyl) amino)benzenesulphonamide (5f)

Yield: 67%; R_f_: 0.47 (chloroform:methanol 9:1); M.P.: 159–162 °C; purity (HPLC): 100%; ^1^H-NMR (300 MHz, DMSO-d_6_) δ ppm: 7.88–7.85 (dd, 2H, Ar–H), 7.57–7.51 (m, 5H, Ar–H), 7.29–7.25 (dd, 2H, Ar–H), 7.16 (s, 1H, Ar–H), 6.93 (s, 2H, SO_2_NH_2_), 6.69–6.66 (dd, 2H, Ar–H), 6.63–6.59 (t, 1H, Ar–NH), 5.82–5.77 (dd, 1H, Hx), 4.57–4.38 (dd, 2H, CH_2_), 4.02–3.92 (dd, 1H, Ha), 3.29–3.23 (dd, 1H, Hb); ^13^C-NMR (100 MHz, DMSO-d_6_): δ = 167.29, 154.58, 151.42, 138.82, 135.87, 131.41, 131.24, 130.06, 129.98, 129.4, 129.19, 128.9, 127.89, 127.69, 126.9, 111.69, 58.37, 45.15, 41.1; IR (ATR, cm^−1^): 3267, 3207 (N–H of NH_2_), 3146 (N–H *str*), 3097 (Ar C–H *str*), 2947 (aliphatic C–H *str*), 1670 (C = O *str*), 1195 (S = O *str*), 759 (C–Cl *str*); LC-MS (ESI; M)^+^: 503.40.

#### 4-((2-(3-(4-Chlorophenyl)-5-(2-hydroxyphenyl)-4,5-dihydro-1H-pyrazol-1-yl)-2-oxoethyl) amino)benzenesulphonamide (5g)

Yield: 72%; R_f_: 0.41 (chloroform:methanol 9:1); M.P.: 161–163 °C; purity (HPLC): 98.58%; ^1^H-NMR (300 MHz, DMSO-d_6_) δ ppm: 10.85 (s, 1H, Ar–OH), 7.85–7.78 (m, 3H, Ar–H), 7.55–7.50 (m, 3H, Ar–H), 7.48–7.41 (dd, 3H, Ar–H), 6.92 (s, 2H, SO_2_NH_2_), 6.69–6.66 (dd, 3H, Ar–H), 6.48–6.45 (t, 1H, Ar–NH), 4.35–4.32 (dd, 2H, CH_2_), 4.01–3.99 (dd, 1H, Ha), 3.35 (dd, 1H, Hb); ^13^C-NMR (100 MHz, DMSO-d_6_): δ = 171.95, 151.54, 147.39, 137.25, 134.4, 131.04, 129.16, 128.73, 128.4, 128.17, 127.72, 111.63, 44.44; IR (ATR, cm^−1^): 3566 (OH *str*), 3356, 3300 (N–H of NH_2_), 3259 (N–H *str*), 3072 (Ar C–H *str*), 2924 (aliphatic C–H *str*), 1670 (C = O *str*), 1147 (S = O *str*), 729 (C–Cl *str*).

#### 4-((2-(3-(4-Chlorophenyl)-5-(3,4-dimethoxyphenyl)-4,5-dihydro-1H-pyrazol-1-yl)-2-oxoethyl)amino)benzenesulphonamide (5h)

Yield: 70%; R_f_: 0.61 (chloroform:methanol 9:1); M.P.: 170–173 °C; ^1^H-NMR (300 MHz, DMSO-d_6_) δ ppm: 7.88–7.82 (dd, 2H, Ar–H), 7.57–7.47 (m, 4H, Ar–H), 6.92 (s, 2H, SO_2_NH_2_), 6.87–6.80 (dd, 2H, Ar–H), 6.71–6.65 (dd, 2H, Ar–H), 6.63–6.59 (t, 1H, Ar–NH), 5.82–5.77 (dd, 1H, Hx), 4.57–4.38 (dd, 2H, CH_2_), 4.02–3.92 (dd, 1H), 3.29–3.23 (dd, 1H, Hb); ^13^C-NMR (100 MHz, DMSO-d_6_): δ = 167.18, 154.48, 149.29, 148.54, 135.67, 134.81, 131.16, 130.29, 129.22, 128.92, 127.66, 117.88, 112.31, 111.62, 110.01, 60.43, 56.02, 55.94; IR (ATR, cm^−1^): 3329 (N–H of NH_2_), 3209 (N–H *str*), 3087 (Ar C–H *str*), 2899 (aliphatic C–H *str*), 1689 (C = O *str*), 1153 (S = O *str*), 732 (C–Cl *str*).

#### 4-((2-(3-(4-Chlorophenyl)-5-(thiophen-2-yl)-4,5-dihydro-1H-pyrazol-1-yl)-2-oxoethyl) amino)benzenesulphonamide (5i)

Yield: 68%; R_f_: 0.45 (chloroform:methanol 9:1); M.P.: 148–150 °C; purity (HPLC): 96.06%; ^1^H-NMR (300 MHz, DMSO-d_6_) δ ppm: 7.89–7.87 (dd, 2H, Ar–H), 7.54–7.50 (m, 4H, Ar–H), 7.37–7.35 (dd, 1H, Ar–H), 7.05–7.04 (dd, 1H, Ar–H), 6.94–6.92 (dd, 1H, Ar–H), 6.91 (s, 2H, SO_2_NH_2_), 6.66–6.64 (dd, 2H, Ar–H), 6.57–6.54 (t, 1H, Ar–NH), 5.93–5.89 (dd, 1H, Hx), 4.41–4.28 (dd, 2H, CH_2_), 3.90–3.83 (dd, 1H, Ha), 3.41–3.40 (dd, 1H, Hb); ^13^C-NMR (100 MHz, DMSO-d_6_): δ = 167.3, 154.67, 151.49, 135.8, 131.16, 130.1, 129.31, 129.03,127.66,127.14, 125.48, 125.2, 111.65, 56.14, 45.13, 41.89; IR (ATR, cm^−1^): 3390, 3308 (N–H of NH_2_), 3236 (N–H *str*), 3074 (Ar C–H *str*), 2924 (aliphatic C–H *str*), 1683 (C = O *str*), 1157 (S = O *str*), 738 (C–Cl *str*); LC-MS (ESI; M)^+^: 475.0.

#### 4-((2-(5-(Anthracen-9-yl)-3-(4-chlorophenyl)-4,5-dihydro-1H-pyrazol-1-yl)-2-oxoethyl) amino)benzenesulphonamide (5j)

Yield: 71%; R_f_: 0.62 (chloroform:methanol 9:1); M.P.: 190–192 °C; purity (HPLC): 100%; ^1^H-NMR (300 MHz, DMSO-d_6_) δ ppm: 8.75–7.61 (m, 4H, Ar–H), 8.22–8.02 (m, 4H, Ar–H), 7.59–7.49 (m, 12H, Ar–H), 6.95–6.92 (dd, 3H, Hx, SO_2_NH_2_), 6.75–6.68 (dd, 2H, Ar–H), 6.58–6.55 (t, 1H, Ar–NH), 4.38–4.35 (dd, 2H, CH_2_), 4.01–4.00 (dd, 1H, Ha), 3.35 (dd, 1H, Hb); ^13^C-NMR (100 MHz, DMSO-d_6_): δ = 166.86, 151.57, 146.71, 137.28, 134.37, 131.66, 131.38, 131.15, 131.05, 130.07, 129.93, 129.81, 129.4, 128.74, 128.42, 128.21, 127.82, 127.74, 127.52, 125.89, 125.84, 125.55, 125.45, 111.63, 44.43, 44.17; IR (ATR, cm^−1^): 3342 (N–H of NH_2_), 3277 (N–H *str*), 3051 (Ar C–H *str*), 2862 (aliphatic C–H *str*), 1672 (C = O *str*), 1161 (S = O *str*), 761 (C–Cl *str*).

#### 4-((2-(3-(4-Chlorophenyl)-5-(1H-indol-3-yl)-4,5-dihydro-1H-pyrazol-1-yl)-2-oxoethyl) amino)benzenesulphonamide (5k)

Yield: 73%; R_f_: 0.55 (chloroform:methanol 9:1); M.P.: 174–177 °C; purity (HPLC): 100%; ^1^H-NMR (300 MHz, DMSO-d_6_) δ ppm: 11.23 (s, 1H, Indole–NH), 8.23–8.15 (dd, 2H, Ar–H), 7.73–7.71 (dd, 1H, Ar–H), 7.57–7.55 (dd, 2H, Ar–H), 7.44 (dd, 1H, Ar–H), 7.22–7.18 (t, 2H, Ar–H), 6.90 (s, 2H, SO_2_NH_2_), 6.71–6.65 (t, 3H, Ar–H, Hx), 6.52–6.49 (t, 1H, Ar–NH), 4.37–4.31 (dd, 2H, CH_2_), 3.88–3.86 (dd, 1H, Ha), 3.50–3.43 (dd, 1H, Hb); ^13^C-NMR (100 MHz, DMSO-d_6_): δ = 165.68, 151.6, 141.54, 137.53, 131.47, 131.02, 130.3, 127.77, 124.76, 124.58, 122.93, 121.94, 120.98, 120.71, 112.26, 111.89, 111.75, 111.56, 56.67, 44.06; IR (ATR, cm^−1^): 3174 (N–H of NH_2_), 3113 (N–H *str*), 3041 (Ar C–H *str*), 2820 (aliphatic C–H *str*), 1672 (C = O *str*), 1122 (S = O *str*), 740 (C–Cl *str*).

#### 4-((2-(3-Methyl-5-phenyl-4,5-dihydro-1H-pyrazol-1-yl)-2-oxoethyl)amino) benzene sulphonamide (5l)

Yield: 79%; R_f_: 0.63 (chloroform:methanol 9:1); M.P.: 211– 213 °C; purity (HPLC): 99.16%; ^1^H-NMR (400 MHz, DMSO-d_6_) δ ppm: 7.68–7.61 (dd, 2H, Ar–H), 7.53–7.51 (m, 3H, Ar–H), 7.47–7.45 (dd, 2H, Ar–H), 7.34–7.32 (dd, 1H, H_x_), 7.19–7.08 (dd, 1H, H_b_), 6.95 (_S_, 2H, SO_2_–NH_2_), 6.67–6.64 (dd, 2H, Ar–H), 6.51–6.48 (t, 1H, Ar–NH), 4.27–4.22 (dd, 2H, CH_2_), 3.99–3.95 (dd, 1H, CHH_a_), 2.08 (s, 3H, CH_3_); IR (ATR) cm^−1^: 3404 (N–H *str* of NH_2_), 3302 (N–H *str*), 3078 (aromatic C–H *str*), 2885 (aliphatic C–H *str*), 1693 (C = O *str*), 1610 (C = N), 1143 (S = O *str*); LC-MS (ESI; M + 1)^+^: 373.1.

#### 4-((2-(5-(4-Methoxyphenyl)-3-methyl-4,5-dihydro-1H-pyrazol-1-yl)-2-oxoethyl) amino)benzenesulphonamide (5m)

Yield: 85%; R_f_: 0.65 (chloroform:methanol 9:1); M.P.: 235 –237 °C; purity (HPLC): 98.89%; ^1^H-NMR (400 MHz, DMSO-d_6_) δ ppm: 7.58–7.56 (dd, 2H, Ar–H), 7.50–7.48 (dd, 2H, Ar–H), 7.46–7.44 (dd, 1H, H_x_), 6.95–6.93 (dd, 1H, H_b_), 6.92–6.90 (dd, 2H, Ar–H), 6.8 (_S_, 2H, SO_2_–NH_2_), 6.69–6.67 (dd, 2H, Ar–H), 6.32–6.30 (t, 1H, Ar–NH), 4.26–4.25 (dd, 2H, CH_2_), 4.0–3.99 (dd, 1H, CHH_a_), 3.79 (s, 3H, OCH_3_), 2.08 (s, 3H, CH_3_); IR (ATR) cm^−1^: 3404 (N–H *str* of NH_2_), 3333 (N–H *str*), 3248 (O–C *str*) 3030 (Ar C–H *str*), 2839 (aliphatic C–H *str*), 1660 (C = O *str*), 1604 (C = N), 1153 (S = O *str*); LC-MS (ESI; M + 1)^+^: 403.1.

#### 4-((2-(5-(4-Chlorophenyl)-3-methyl-4,5-dihydro-1H-pyrazol-1-yl)-2-oxoethyl) amino)benzenesulphonamide (5n)

Yield: 85%; R_f_: 0.61 (chloroform:methanol 9:1); M.P.: 221– 225 °C; purity (HPLC): 98.89%; ^1^H-NMR (400 MHz, DMSO-d_6_) δ ppm: 7.61–7.54 (dd, 2H, Ar–H), 7.54–7.51 (dd, 2H, Ar–H), 7.43–7.33 (dd, 2H, Ar–H), 7.01–6.99 (dd, 1H, H_x_), 6.97–6.93 (dd, 1H, H_b_), 6.87 (_S_, 2H, SO_2_–NH_2_), 6.69–6.67 (dd, 2H, Ar–H), 6.32–6.30 (t, 1H, Ar–NH), 4.26–4.25 (dd, 2H, CH_2_), 3.99–3.40 (dd, 1H, CHH_a_), 2.1 (s, 3H, CH_3_); IR (ATR) cm^−1^: 3408 (N–H *str* of NH_2_), 3288 (N–H *str*), 2885 (aliphatic C–H *str*), 1697 (C = O *str*), 1608 (C = N), 1145 (S = O *str*), 810 (C–Cl *str*); LC-MS (ESI; M + 1)^+^: 407.1.

#### 4-((2-(3-Methyl-5-(p-tolyl)-4,5-dihydro-1H-pyrazol-1-yl)-2-oxoethyl) amino)benzene sulphonamide (5o)

Yield: 78%; R_f_: 0.7 (chloroform:methanol 9:1); M.P.: 233–235 °C; purity (HPLC): 99.46%; ^1^H-NMR (400 MHz, DMSO-d_6_) δ ppm: 7.76–7.75 (dd, 2H, Ar–H), 7.57–7.55 (dd, 2H, Ar–H), 7.35–7.34 (dd, 1H, H_x_), 7.11–7.09 (dd, 2H, Ar–H), 6.87 (_S_, 2H, SO_2_–NH_2_), 6.84–6.81 (dd, 1H, H_b_), 6.64–6.60 (t, 1H, Ar–NH), 6.62–6.60 (dd, 2H, Ar–H), 4.24–4.23 (dd, 2H, CH_2_), 3.97–3.96 (dd, 1H, CHH_a_), 2.27 (s, 3H, Ar–CH3), 2.04 (s, 3H, CH_3_); IR (ATR) cm^−1^: 3319 (NH *str* of NH2), 3232 (NH *str*), 3113 (Ar C–H *str*), 2916 (aliphatic C–H *str*), 1668 (C = O *str*), 1600 (C = N), 1151 (S = O *str*); LC-MS (ESI; M + 1)^+^: 387.1.

#### 4-((2-(5-(4-Fluorophenyl)-3-methyl-4,5-dihydro-1H-pyrazol-1-yl)-2-oxoethyl) amino)benzenesulphonamide (5p)

Yield: 76%; R_f_: 0.81 (chloroform:methanol 9:1); M.P.: 217–219 °C; purity (HPLC): 99.67%; ^1^H-NMR (400 MHz, DMSO-d_6_) δ ppm: 7.68–7.64 (dd, 2H, Ar–H), 7.53–7.51 (dd, 2H, Ar–H), 7.28–7.22 (dd, 2H, Ar–H), 7.12–7.08 (dd, 1H, H_x_), 6.94 (_S_, 2H, SO_2_–NH_2_), 6.68–6.66 (dd, 1H, H_b_), 6.68–6.66 (dd, 2H, Ar–H), 6.52–6.49 (t, 1H, Ar–NH), 4.27–4.25 (dd, 2H, CH_2_), 3.99–3.97 (dd, 1H, CHH_a_), 2.1 (s, 3H, CH_3_); IR (ATR) cm^−1^: 3406 (NH *str* of NH2), 3294 (NH *str*), 3051 (Ar C–H *str*), 2901 (aliphatic C–H *str*), 1693 (C = O *str*), 1600 (C = N), 1307 (C–F *str*), 1145 (S = O *str*); LC-MS (ESI; M + 1)^+^: 391.23.

#### 4-((2-(5-(2-Chlorophenyl)-3-methyl-4,5-dihydro-1H-pyrazol-1-yl)-2-oxoethyl) amino)benzenesulphonamide (5q)

Yield: 76%; R_f_: 0.64 (chloroform:methanol 9:1); M.P.: 202–205 °C; purity (HPLC): 99.40%; ^1^H-NMR (400 MHz, DMSO-d_6_) δ ppm: 7.54–7.52 (dd, 2H, Ar–H), 7.47–7.41 (dd, 1H, H_x_), 7.37–7.28 (m, 4H, Ar–H), 7.00–6.94 (dd, 1H, H_b_), 6.90 (_S_, 2H, SO_2_–NH_2_), 6.68–6.66 (dd, 2H, Ar–H), 6.46–6.43 (t, 1H, Ar–NH), 4.27–4.25 (dd, 2H, CH_2_), 4.0–3.9 (dd, 1H, CHH_a_), 2.1 (s, 3H, CH_3_); IR (ATR) cm^−1^: 3406 (NH *str* of NH2), 3279 (NH *str*), 3064 (Ar C–H *str*), 2885 (aliphatic C–H *str*), 1693 (C = O *str*), 1610 (C = N), 1141 (S = O *str*), 746 (C–Cl *str*); LC-MS (ESI; M + 1)^+^: 407.1.

#### 4-((2-(5-(2-Hydroxyphenyl)-3-methyl-4,5-dihydro-1H-pyrazol-1-yl)-2-oxoethyl) amino)benzenesulphonamide (5r)

Yield: 86%; R_f_: 0.59 (chloroform:methanol 9:1); M.P.: 211–213 °C; purity (HPLC): 99.35%; ^1^H-NMR (400 MHz, DMSO-d_6_) δ ppm: 9.69 (_S_, 1H, Ar–OH), 7.55–7.53 (dd, 2H, Ar–H), 7.50–7.48 (dd, 1H, Ar–H), 7.25–7.2 (dd, 1H, Ar–H), 7.09–7.05 (dd, 1H, Ar–H), 6.97–6.92 (dd, 1H, H_x_), 6.87–6.856 (dd, 1H, H_b_), 6.85 (_S_, 2H, SO_2_–NH_2_), 6.80–6.76 (dd, 1H, Ar–H), 6.67–6.65 (dd, 2H, Ar–H), 6.34–6.31 (t, 1H, Ar–NH), 4.24–4.23 (dd, 2H, CH_2_), 3.97–3.96 (dd, 1H, CHH_a_), 2.1 (s, 3H, CH_3_); IR (ATR) cm^−1^: 3336 (NH *str*), 3252 (OH *str*), 2893 (aliphatic C–H *str*), 1658 (C = O *str*), 1600 (C = N), 1153 (S = O *str*); LC-MS (ESI; M + 1)^+^: 389.1.

#### 4-((2(5-(4-Hydroxyphenyl)-3-methyl-4,5-dihydro-1H-pyrazol-1- yl)-2-oxoethyl) amino)benzenesulphonamide (5s)

Yield: 77%; R_f_: 0.50 (chloroform:methanol 9:1);M.P.: 243–246 °C; purity (HPLC): 99.57%; ^1^H-NMR (400 MHz, DMSO-d_6_) δ ppm: 9.52 (_S_, 1H, Ar–OH), 7.55–7.53 (dd, 2H, Ar–H), 7.36–7.3 (dd, 2H, Ar–H), 6.92–6.86 (dd, 1H, H_b_), 6.84 (_S_, 2H, SO_2_–NH_2_), 6.76–6.74 (dd, 2H, Ar–H), 6.67–6.64 (dd, 2H, Ar–H), 6.54–6.53 (dd, 1H, H_x_), 6.52–6.49 (t, 1H, Ar–NH), 4.23–4.21 (dd, 2H, CH_2_), 3.96–3.95 (dd, 1H, CHH_a_), 2.06 (s, 3H, CH_3_); IR (ATR) cm^−1^: 3389 (NH *str* of NH2), 3315 (NH *str*), 3263 (OH *str*), 3020 (Ar C–H *str*), 2902 (aliphatic C–H *str*), 1680 (C = O *str*), 1602 (C = N), 1143 (S = O *str*); LC-MS (ESI; M + 1)^+^: 389.1.

#### 4-((2-(5-(4-(Dimethylamino)phenyl)-3-methyl-4,5-dihydro-1Hpyrazol-1-yl)-2-oxoethyl) amino)benzenesulphonamide) (5t)

Yield: 86%; R_f_: 0.7 (chloroform:methanol 9:1); M.P.: 239–240 °C; purity (HPLC): 99.26%; ^1^H-NMR (400 MHz, DMSO-d_6_) δ ppm: 7.53–7.51 (dd, 2H, Ar–H), 7.43–7.41 (dd, 2H, Ar–H), 6.99–6.95 (dd, 1H, H_b_), 6.93 (_S_, 2H, SO_2_–NH_2_), 6.72–6.70 (dd, 2H, Ar–H), 6.72–6.70 (dd, 1H, H_x_), 6.68–6.66 (dd, 2H, Ar–H), 6.50–6.47 (t, 1H, Ar–NH) , 4.25–4.24 (dd, 2H, CH_2_), 3.97–3.95 (dd, 1H, CHH_a_), 2.07 (s, 3H, CH_3_); IR (ATR) cm^−1^: 3325 (N–H *str* of NH2), 3236 (N–H *str*), 3107 (Ar C–H *str*), 2899 (aliphatic C–H *str*), 1660 (C = O *str*), 1602 (C = N *str*), 1311 (C–N ter. amine *str*), 1238 (C–N aromatic *str*) 1153 (S = O *str*); LC-MS (ESI; M + 1)^+^: 416.1.

#### 4-((2-(3-Methyl-5-(thiophen-2-yl)-4,5-dihydro-1H-pyrazol-1-yl)-2-oxoethyl) amino)benzenesulphonamide (5u)

Yield: 82%; R_f_: 0.63 (chloroform:methanol 9:1); M.P.: 197–199 °C; purity (HPLC): 99.099%; ^1^H-NMR (400 MHz, DMSO-d_6_) δ ppm: 7.55–7.53 (dd, 2H, Ar–H), 7.38–7.37 (dd, 1H, Ar–H), 7.13–7.08 (dd, 1H, Ar–H), 7.08–7.06 (dd, 1H, H_b_), 7.01–7.00 (dd, 1H, Ar–H), 6.83 (_S_, 2H, SO_2_–NH_2_), 6.66–6.64 (dd, 2H, Ar–H), 6.46–6.45 (dd, 1H, H_x_), 6.26 (t, 1H, Ar–NH), 4.23–4.22 (dd, 2H, CH_2_), 3.97–3.96 (dd, 1H, CHH_a_), 2.05 (s, 3H, CH_3_); IR (ATR) cm^−1^: 3402 (NH *str* of NH2), 3296 (NH *str*), 3074 (Ar C–H *str*), 2881 (aliphatic C–H *str*), 1691 (C = O *str*), 1600 (C = N), 1143 (S = O *str*); LC-MS (ESI; M + 1)^+^: 379.1.

#### 4-((2-(2-Benzylidenehydrazinyl)-2-oxoetehyl) amino) benzenesulphonamide (6a)

Yield: 80%; R_f_: 0.69; M.P.: 223–226 °C; ^1^H-NMR (400 MHz, DMSO-d_6_) δ ppm: 11.51 (s, 1H, NH), 8.02 (s, 1H, CH), 7.72–7.67 (m, 4H), 7.45–7.38 (m, 5H), 6.87 (s, 2H, NH_2_), 6.32 (t, 1H, NH), 4.33 (d, 2H, CH_2_); ^13^C-NMR (400 MHz, DMSO-d_6_) δ ppm: 43.49 (CH_2_), 170.60 (C = O), 143.67 (N = CH); IR (ATR) cm^−1^: 3313 (N–H *str* of NH_2_), 3255 (N–H *str*), 3173 (Ar C–H *str*), 3070 (aliphatic C–H *str*), 1681 (C = N *str*), 1145 (S = O *str*); LC-MS (ESI; M + 1)^+^: 333.2.

#### 4-((2-(2-(4-Methylbenzylidene) hydrazinyl)-2-oxoethyl)amino)benzene sulphonamide (6b)

Yield: 85%; R_f_: 0.75; M.P.: 220–225 °C. ^1^H-NMR (400 MHz, DMSO-d_6_) δ ppm: 11.44 (s, 1H, NH), 7.98 (s, 1H, CH), 7.59–7.57 (m, 4H), 7.23–7.19 (dd, 2H), 6.86 (s, 2H, NH_2_), 6.70–6.65 (dd, 2H), 6.30 (t,1H, NH), 4.29 (d, 2H, CH_2_), 2.39 (s,3H, CH_3_); ^13^C-NMR (400 MHz, DMSO-d_6_) δ ppm: 43.47 (CH_2_), 170.44 (C = O), 143.78 (N = CH), 21.03 (CH_3_); IR (ATR) cm^−1^: 3319 (N–H *str* of NH_2_), 3257 (N–H *str*), 3194 (Ar C–H *str*), 3078 (aliphatic C–H *str*), 1683 (C = N *str*), 1145 (S = O *str*); LC-MS (ESI; M)^+^: 346.9.

#### 4-((2-(2-(2-Hydroxybenzylidene)hydrazinyl)-2-oxoethyl) amino) benzenesulphonamide (6c)

Yield: 85%; R_f_: 0.82; M.P.: 213–216 °C. ^1^H-NMR (400 MHz, DMSO-d_6_) δ ppm: 11.50 (s, 1H, NH), 10.01 (s, 1H, OH), 8.31 (s, 1H, CH), 7.58–7.54 (dd, 2H), 7.45–7.43 (d,1H), 7.27–7.21 (d,1H), 6.86 (s, 2H, NH_2_), 6.69–6.65 (m, 4H), 6.41 (t, 1H, NH), 4.29 (d, 2H, CH_2_); ^13^C-NMR (400 MHz, DMSO-d_6_) δ ppm: 43.48 (CH_2_), 170.10 (C = O), 144.10 (N = CH); IR (ATR) cm^−1^: 3443 (O-H *str*), 3369 (N–H *str* of NH_2_), 3265 (N–H *str*), 3113 (Ar C–H *str*), 3093 (aliphatic C–H *str*), 1681 (C = N *str*), 1120 (S = O *str*); LC-MS (ESI; M)^+^: 348.3.

#### 4-((2-Oxo-2-(2-(pyridin-2-ylmethylene) hydrazinyl) ethyl) amino) benzenesulphonamide (6d)

Yield 80%; R_f_: 0.78; M.P.: 245–250 °C. ^1^H-NMR (400 MHz, DMSO-d_6_) δ ppm: 11.74 (s, 1H, NH), 8.02 (s, 1H, CH), 7.95–7.93 (m, 4H), 7.86–7.79 (d, 1H), 7.39–7.36 (d, 1H), 6.91 (s, 2H, NH_2_), 6.46 (t, 1H, NH), 4.36 (d, 2H, CH_2_); ^13^C-NMR (400 MHz, DMSO-d_6_) δ ppm: 43.50 (CH_2_), 170.82 (C = O), 144.19 (N = CH); IR (ATR) cm^−1^: 3354 (N–H *str* of NH_2_), 3236 (N–H *str*), 3097 (Ar C–H *str*), 3072 (aliphatic C–H *str*), 1685 (C = N *str*), 1145 (S = O *str*); LC-MS (ESI; M + 1)^+^: 334.3.

#### 4-((2-(2-(3,4-Dimethoxybenzylidene)hydrazinyl)-2-oxoethyl)amino) benzenesulphonamide (6e)

Yield: 86%; R_f_: 0.84; M.P.: 230–233 °C. ^1^H-NMR (400 MHz, DMSO-d_6_) δ ppm: 11.40 (s, 1H, NH), 7.94 (s, 1H, CH), 7.57–7.54 (m, 4H), 7.20–6.94 (m, 3H), 6.88 (s, 2H, NH_2_), 6.69 (t, 1H, NH), 4.31 (d, 2H, CH_2_), 3.85 (s, 6H, 2-OCH_3_); ^13^C-NMR (400 MHz, DMSO-d_6_) δ ppm: 43.48 (CH_2_), 170.28 (C = O), 143.46 (N = CH), 55.11 (2O–CH_3_); IR (ATR) cm^−1^: 3306 (N–H *str* of NH_2_), 3248 (N–H *str*), 3171 (Ar C–H *str*), 3086 (aliphatic C–H *str*), 1683 (C = N *str*), 1143 (S = O *str*), 1255 (C–O *str*); LC-MS (ESI; M + 1)^+^: 393.29.

#### 4-((2-(2-(4-Methoxybenzylidene) hydrazinyl)-2-oxoethyl) amino) benzenesulphonamide (6f)

Yield: 85%; R_f_: 0.79; M.P.: 240–245 °C. ^1^H-NMR (400 MHz, DMSO-d_6_) δ ppm: 11.40 (s, 1H, NH), 7.96 (s, 1H, CH), 7.66–7.61 (m, 4H), 6.99–6.95 (dd, 2H), 6.90 (s, 2H, NH_2_), 6.70–6.65 (dd, 2H), 6.43–6.41 (t,1H, NH), 4.29 (d, 2H, CH_2_), 3.81 (s, 3H, OCH_3_); ^13^C-NMR (400 MHz, DMSO-d_6_): δ ppm 43.52 (CH_2_), 170.37 (C = O), 143.71 (N = CH), 55.49 (O–CH_3_); IR (ATR) cm^−1^: 3294 (N–H *str* of NH_2_), 3275 (N–H *str*), 3198 (Ar C–H *str*), 3099 (aliphatic C–H *str*), 1680 (C = N*str*), 1143 (S = O *str*); LC-MS (ESI; M)^+^: 362.9.

#### 4-((2-(2-(4-Hydroxybenzylidene)hydrazinyl)-2-oxoethyl) amino) benzenesulphonamide (6g)

Yield 80%; R_f_: 0.81; M.P.: 214–218 °C. ^1^H-NMR (400 MHz, DMSO-d_6_) δ ppm: 11.25 (s, 1H, NH), 9.73 (s, 1H, OH), 7.85 (s, 1H, CH), 7.50–7.43 (m, 4H), 6.77–6.72 (dd, 2H), 6.63–6.60 (dd, 2H), 6.82 (s, 2H, NH_2_), 6.58 (t, 1H, NH), 4.20 (d, 2H, NH); ^13^C-NMR (400 MHz, DMSO-d_6_) δ ppm: 43.48 (CH_2_), 170.10 (C = O), 144.10 (N = CH); IR (ATR) cm^−1^: 3392 (O-H *str*), 3313 (N–H *str* of NH_2_), 3292 (N–H *str*), 3211 (Ar C–H *str*), 3022 (aliphatic C–H *str*), 1685 (C = N *str*), 1136 (S = O *str*); LC-MS (ESI; M + 1)^+^: 349.3.

#### 4-((2-(2-(4-Hydroxy-3,5-dimethoxybenzylidene)hydrazinyl)-2-oxoethyl)amino) benzenesulphonamide (6h)

Yield: 85%; R_f_: 0.85; M.P.: 255–260 °C. ^1^H-NMR (400 MHz, DMSO-d_6_) δ ppm: 11.33 (s, 1H, NH), 9.83 (s, 1H, OH), 7.82 (s, 1H, CH), 7.51–7.47 (dd, 2H), 6.83–6.81 (dd, 2H), 6.88 (s, 2H, NH_2_), 6.63–6.56 (s,2H), 6.35 (t,1H, NH), 4.26 (d, 2H, CH_2_), 3.78 (s, 6H, 2OCH_3_); ^13^C-NMR (400 MHz, DMSO-d_6_) δ ppm: 43.48 (CH_2_), 170.29 (C = O), 147.98 (N = CH), 55.95 (O–CH_3_); IR (ATR) cm^−1^: 3408 (O-H *str*), 3304 (N–H *str* of NH_2_), 3279 (N–H *str*), 3196 (Ar C–H *str*), 3106 (aliphatic C–H *str*), 1685 (C = N *str*), 1143 (S = O *str*), 1219 (C–O *str*), LC-MS (ESI; M + 1)^+^: 409.3.

#### 4-((2-Oxo-2-(2-(3,4,5-trimethoxybenzylidene) hydrazinyl)ethyl) amino) benzenesulphonamide (6i)

Yield: 80%; R_f_: 0.80; M.P.: 250–253 °C. ^1^H-NMR (400 MHz, DMSO-d_6_) δ ppm: 11.33 (s, 1H, NH), 7.82 (s, 1H, CH), 7.51–7.47 (dd, 2H), 6.83–6.81 (dd, 2H), 6.88 (s, 2H, NH_2_), 6.63–6.56 (dd, 2H), 6.35 (t, 1H, NH), 4.26 (d, 2H, CH_2_), 3.78 (s, 6H, 2OCH_3_), 3.82 (s, 3H, OCH_3_); ^13^C-NMR (400 MHz, DMSO-d_6_) δ ppm: 40.21 (CH_2_), 170.47 (C = O), 143.62 (N = CH), 55.11 (O–CH_3_), 60.03 (2O–CH_3_); IR (ATR) cm^−1^: 3304 (N–H *str* of NH_2_), 3271 (N–H *str*), 3201 (Ar C–H *str*), 3107 (aliphatic C–H *str*), 1683 (C = N*str*), 1144 (S = O *str*), 1236 (C–O *str*); LC-MS (ESI; M + 1)^+^: 423.3.

#### 4-((2-(2-(2,3-Dimethoxybenzylidene)hydrazinyl)-2-oxoethyl)amino)benzenesulphonamide (6j)

Yield: 80%; R_f_: 0.77; M.P.: 240–244 °C. ^1^H-NMR (400 MHz, DMSO-d_6_) δ ppm: 11.40 (s, 1H, NH), 7.94 (s, 1H, CH), 7.57–7.54 (dd, 2H), 7.32–7.31 (dd, 2H), 7.20–6.94 (m, 3H), 6.88 (s, 2H, NH_2_), 6.69–6.65 (t, 1H, NH), 4.31 (d, 2H, CH_2_), 3.85 (s, 6H, OCH_3_); ^13^C-NMR (400 MHz, DMSO-d_6_) δ ppm: 43.48 (CH_2_), 170.28 (C = O), 143.46 (N = CH), 55.11 (2O–CH_3_); IR (ATR) cm^−1^: 3387 (N–H *str* of NH_2_), 3255 (N–H *str*), 3186 (Ar C–H *str*), 3070 (aliphatic C–H *str*), 1687 (C = N*str*), 1172 (S = O *str*), 1230 (C–O *str*); LC-MS (ESI; M)^+^: 392.9.

### 2.2. CA activity/inhibition studies

An Sx.18Mv-R Applied Photophysics (Oxford, UK) stopped-flow instrument has been used to assay the catalytic activity of various CA isozymes for CO_2_ hydration reaction^20^. Phenol red (at a concentration of 0.2 mM) was used as indicator, working at the absorbance maximum of 557 nm, with 10 mM Hepes (pH 7.5) or 10 mM Tris (pH 8.5) as buffers, and 0.1 M Na_2_SO_4_ (for maintaining constant ionic strength, which is not inhibitory against these enzymes), following the CA-catalysed CO_2_ hydration reaction for a period of 10 s at 25 °C. The CO_2_ concentrations ranged from 1.7 to 17 mM for the determination of the kinetic parameters and activation constants. For each inhibitor at least six traces of the initial 5–10% of the reaction have been used for determining the initial rate. The uncatalysed rates were determined in the same manner and subtracted from the total observed rates. Stock solutions of inhibitors (10 mM) were prepared in distilled-deionised diluted to 1 nM using the assay buffer. Inhibitor and enzyme solutions were pre-incubated together for 15 min (standard assay at room temperature) prior to assay, in order to allow for the formation of the enzyme–inhibitor complex. The inhibition constant (K_I_), was obtained by considering the classical Michaelis–Menten equation and the Cheng-Prusoff algorithm by using non-linear least squares fitting as reported earlier^21–24^.

## 3. Results and discussion

### 3.1. Chemistry

**Scheme 1.**
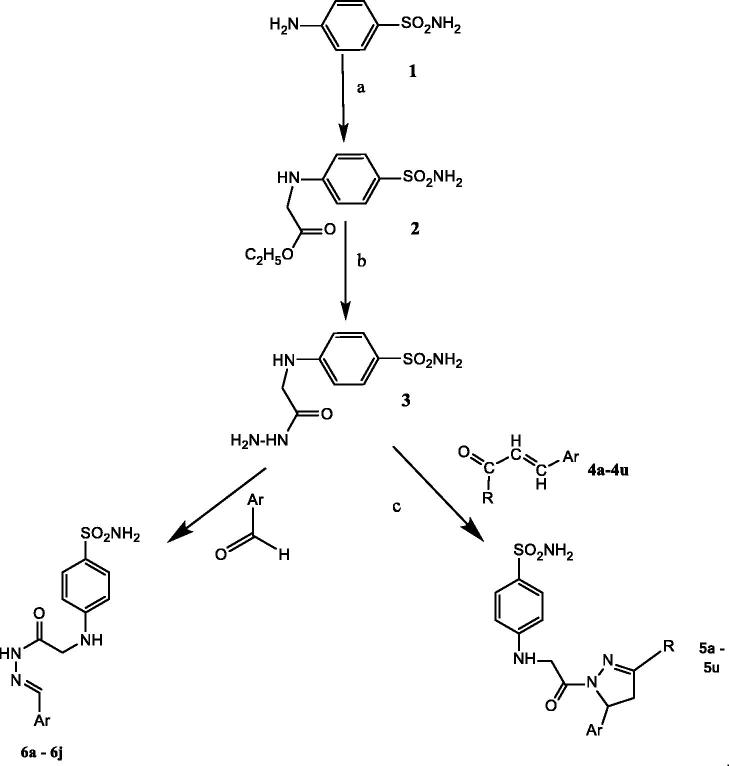
Synthesis of compounds **5a–5u** and **6a–6j.** Reagents and conditions were: (a) Br–CH_2_COOC_2_H_5_, K_2_CO_3,_ EtOH, reflux, 12 h; (b) NH_2_NH_2_·H_2_O, EtOH, reflux, 4–6 h. (c) For compounds **5a–5k** EtOH, KOH, reflux, 4 h, overnight stirring and for **5l–5u** glacial acetic acid, reflux.

The key intermediate **1** (ethyl 2-((4-sulphamoylphenyl) amino) acetate) was obtained in good yields by the reaction of sulphanilamide with ethyl bromoacetate in the presence of potassium carbonate (no sulphonamide N-alkylation occurred), Scheme 1. During optimisation, the same reaction was performed with sodium carbonate instead of potassium carbonate. The results were not satisfactory, and whence the equimolar ratio of both the reactants in the presence of K_2_CO_3_ and ethanol as a solvent was used to obtain compound **1**. 4-((2-Hydrazinyl-2-oxoethyl)amino) benzene sulphonamide (**2**) was obtained by the reaction of hydrazine hydrate with **1** in equimolar ratios. The reaction was refluxed for 3 h at 70 °C producing the compound with yields of 70–72%. Reaction of **2** with various substituted aromatic aldehydes afforded derivatives **6**. When using unsaturated aldehydes, a cyclisation reaction occurred after the Schiff base formation, leading to compounds **5** (Scheme 1).

The structures of 4-((2-(3-alky/aryl-5-ary/heteroaryl-4,5-dihydro-1H-pyrazol-1-yl)-2-oxoethyl)amino)benzenesulphonamides **5a–5u** were confirmed by mass spectrometry (MS), Fourier-transform infrared spectroscopy (FTIR) and ^1^H and ^13^C-nuclear magnetic resonance (NMR). The structures of compounds were analysed by FTIR spectra which revealed that N–H stretches of amines in the region 3500–3000 cm^−1^. Spectra revealed presence of C = O stretching vibrations of amide in the region 1695–1630 cm^−1^, aliphatic C–H stretching vibration was observed in 2920–2800 cm^−1^ and C = N stretching vibration was observed at 1615–1564 cm^−1^. Further evidence for formation of target compound was obtained from ^1^H-NMR spectra which provided diagnostic tool for the positional elucidation of the protons. The Ar–NH proton was appeared at δ = 6.58 ppm as triplet. The formed pyrazoline was confirmed with doublet of doublet for CH_2_ giving signal δ = 4.0–3.8 ppm (H_a_), H_b_ at δ 7.1–6.6 ppm (compounds **5l–5u**) and at δ = 3.26–3.13 ppm (compounds **5a–5k**), CH giving signal at δ = 7.4–7.1 ppm (H_x_ of series 5l–5u) and δ = 5.61–5.56 ppm (H_x_ of series **5a–5k**). The characteristic doublet signal of aromatic protons was observed between δ = 7.9 and 6.6 ppm. The NH protons of SO_2_–NH_2_ as singlet between δ = 6.94 and 6.80 ppm was observed. Singlet in the range of δ = 6.59–6.2 ppm for Ar–NH protons was observed. The characteristic singlet signal of CH_3_ protons in series 5l–5u was observed between δ = 2.1 and 2.04 ppm. Mass spectroscopy was done for newly synthesised compounds. The base peak *m/z* for the compounds were found as (M + 1)^+^ with respective to their molecular weight except for 5i it is was found to be (M)^+^.

High-performance liquid chromatography (HPLC) was done for newly synthesised compounds. Using area normalisation method, the percent purity for the compounds was found to be above 88%. The structures of novel Schiff bases **6a–6j** were confirmed by MS, FTIR and ^1^H and ^13^C-NMR. The IR spectra displayed an intense absorption band in the range of 1615–1630 cm^−1^, characteristic of the carbonyl groups. Additionally, intense bands, originating from the stretching vibration of the C = N group of the azomethine were observed at 1685 and 3313 cm^−1^ for N–H *str* of NH_2_ following the aliphatic C–H *str* displayed vibration at 3022 cm^−1^. Further, we observed ^1^H and ^13^C-NMR interpretation for singlet peak at chemical shift δ range in 7.95–8.02 ppm and 143.71–144.10 ppm, confirming the presence of azomethine group in the compound, respectively (Tables 1 and 2).

**Table 1.** Physicochemical properties of 4-((2-(3-alky/aryl-5-ary/heteroaryl-4,5-dihydro-1H-pyrazol-1-yl)-2-oxoethyl)amino)benzenesulphonamide derivatives (**5a–5u)**.


Compound code	R	Ar	Molecular formula	Mol. Wt.	Yield (%)
**5a**	p-Cl-C_6_H_4_	Phenyl	C_23_H_21_N_4_O_3_SCl	468.10	70
**5b**		4-Fluorophenyl	C_23_H_20_N_4_O_3_SClF	486.09	68
**5c**		4-Bromophenyl	C_23_H_20_N_4_O_3_SClBr	547.85	75
**5d**		4-Methylphenyl	C_24_H_23_N_4_O_3_SCl	482.12	70
**5e**		Pyridin-4-yl	C_22_H_20_N_5_O_3_SCl	469.94	71
**5f**		2-Chlorophenyl	C_23_H_20_N_4_O_3_SCl_2_	503.40	67
**5g**		2-Hydroxyphenyl	C_23_H_21_N_4_O_4_SCl	484.96	72
**5h**		3,4-Dimethoxy phenyl	C_25_H_25_N_4_O_5_SCl	529.01	70
**5i**		Thiophen-2-yl	C_21_H_19_N_4_O_3_S_2_Cl	474.98	68
**5j**		Anthran-9-yl	C_31_H_25_N_4_O_3_SCl	569.13	71
**5k**		Indol-3-yl	C_25_H_22_N_5_O_3_SCl	507.99	73
**5l**	Me	Phenyl	C_18_H_20_N_4_O_3_S	372.44	79
**5m**		4-Methoxyphenyl	C_19_H_22_N_4_O_4_S	402.46	85
**5n**		4-Chlorophenyl	C_18_H_19_N_4_O_3_SCl	406.88	85
**5o**		4-Methylphenyl	C_19_H_22_N_4_O_3_S	386.46	78
**5p**		4-Fluorophenyl	C_18_H_19_N_4_O_3_SF	390.43	76
**5q**		2-Chlorophenyl	C_18_H_19_N_4_O_3_SCl	406.88	76
**5r**		2-Hydroxyphenyl	C_18_H_20_N_4_O_4_S	388.44	86
**5s**		4-Hydroxyphenyl	C_18_H_20_N_4_O_4_S	388.44	77
**5t**		4-(Dimethyl)amine phenyl	C_20_H_25_N_5_O_3_S	415.5	86
**5u**		2-Thienyl	C_16_H_18_N_4_O_3_S_2_	378.46	82

**Table 2.** Physicochemical properties of 4-((2-(arylmethylidene)hydrazinyl)-2-oxoethyl)amino) benzene sulphonamide derivatives (**6a–6j**).


Compound code	Ar	Molecular Formula	Molecular weight	Yield (%)
**6a**	Phenyl	C_15_H_16_N_4_O_3_S	332.38	80
**6b**	4-Methylphenyl	C_16_H_18_N_4_O_3_S	346.40	85
**6c**	2-Hydroxyphenyl	C_15_H_16_N_4_O_4_S	348.38	85
**6d**	Pyridin-2-yl	C_14_H_15_N_5_O_3_S	333.37	80
**6e**	3,4-Dimethoxyphenyl	C_17_H_20_N_4_O_5_S	392.43	86
**6f**	4-Methoxyphenyl	C_16_H_18_N_4_O_4_S	362.40	85
**6g**	4-Hydroxyphenyl	C_15_H_16_N_4_O_4_S	348.38	80
**6h**	4-Hydroxy-3,5-dimethoxy	C_17_H_20_N_4_O_6_S	408.43	85
**6i**	3,4,5-Trimethoxy phenyl	C_18_H_22_N_4_O_6_S	422.46	80
**6j**	2,3-Dimethoxyphenyl	C_17_H_20_N_4_O_5_S	392.43	80

### 3.2. CA inhibition

The compounds **5** and **6** reported here were investigated as inhibitors of three CAs involved in crucial physiologic processes and known to act as drug targets, i.e. the human (h) isoforms hCA I and II (belonging to the α-CA class) and the bacterial enzyme mtCA3 from *Mycobacterium tuberculosis* (a β-class CA) (Table 3). Acetazolamide (AAZ), a clinically used sulphonamide has been employed as standard inhibitor in the assay.

**Table 3.** hCA I, II and mtCA 3 inhibition data of compounds **5** and **6** reported in the article, by a stopped-flow CO_2_ hydrase assay^20^.

Compound	K_i_ (nM)^a^		
	hCA I	hCA II	mtCA 3
**5a**	306	282	1800
**5b**	441	500	486
**5c**	634	2850	732
**5d**	93.4	426	175
**5e**	175	42.3	138
**5f**	445	433	215
**5g**	174	578	147
**5h**	280	424	186
**5i**	276	373	233
**5j**	91.8	2020	157
**5k**	440	392	127
**5l**	760	810	1583
**5m**	2374	5545	>10,000
**5n**	835	944	1288
**5o**	898	3461	275
**5p**	665	903	250
**5q**	742	648	623
**5r**	721	83.1	1592
**5s**	738	73.6	265
**5t**	842	5154	1735
**5u**	798	126	1452
**6a**	220	549	>10,000
**6b**	266	914	>10,000
**6c**	54.6	32.1	2145
**6d**	316	418	>10,000
**6e**	1802	758	736
**6f**	648	703	2128
**6g**	327	255	254
**6h**	292	483	2003
**6i**	890	446	216
**6j**	785	240	232
**AAZ**	250	12.1	104

AAZ was used as standard drug.

^a^Mean from three different assays. The errors were in the range of ±10% of the reported values.

As seen from data of Table 3, all investigated compounds inhibited the three enzymes, but generally with a medium potency. Thus, the inhibition constants (K_I_s) were in the range of 54.6 nM–1.8 µM against hCA I, in the range of 32.1 nM–5.5 µM against hCA II and of 127 nM–2.12 µM against mtCA 3, showing a quite flat structure–activity relationship, except for some particular cases which will be discussed in detail.

Thus, for hCA I, the best inhibitors were **5d, 5j** and **6c**, with K_I_s ranging between 54.6 and 93.4 nM, being thus much better inhibitors compared to the standard acetazolamide (K_I_ of 250 nM, Table 3). These compounds incorporate *p*-chlorophenyl and *p*-tolyl moieties (**5d**), *p*-chlorophenyl and 9-anthranyl (**5j**) moieties, and the 2-hydroxyphenyl fragment in the case of **6c**, which are in fact not very different from those found in compounds showing a much worse inhibitory pattern (e.g. **5c**, **5k**, **6e**, etc.). Thus, the explanation that we propose is that the quite long and flexible linker between the benzenesulphonamide fragment and the imine or heterocylic parts of the molecule, affords for a multitude of diverse orientation of the tail present in these compounds, which is probably detrimental to a tight binding, except for the few cases mentioned above, i.e. **5d**, **5j** and **6c**, for which probably some of these conformations assure good interactions with the enzyme active site. However, for the majority of these derivatives, these various conformations/orientations may be not favourable, which explain why most of them have inhibition constants in the high nanomolar–micromolar range (Table 3).

More or less the same situation was observed for the inhibition of hCA II, but for this isoform the most effective inhibitors were **5e**, **5r**, **5s** and **6c**, with K_I_s ranging between 32.1 and 83.1 nM (Table 3). Compound **5e** is the only derivative incorporating a 4-pyridyl moiety, which seems to be effective in inducing strong hCA II inhibitory effects, whereas the remaining ones incorporate 2- or 4-hydroxyphenyl groups. However, the change of these groups to halogens or to methoxy leads to a strong loss of inhibitory effects. The explanation we propose is the same as above for the discussion of hCA I inhibition data.mtCA3 was also effectively inhibited by several of the new compounds, such as **5e, 5g, 5j a**nd **5k**, which showed K_I_s ranging between 127 and 157 nM (acetazolamide has an inhibition constant of 104 nM, being only slightly more effective compared to these sulphonamides). However, the largest majority of these derivatives showed K_I_s in the range of 250 nM–2.12 µM, being thus much less effective inhibitors.

## 4. Conclusion

We report here a new series of sulphonamide derivatives, which was obtained by reaction of a hydrazide derivative with aromatic/heterocyclic aldehydes, followed by an eventual cyclisation to a five-membered heterocylic system. The compounds were designed to incorporate moieties known to induce effective inhibitory for CA isoforms involved in crucial physiologic or pathologic processes such as the cytosolic hCA I and hCA II and the bacterial enzyme mtCA3 from *Mycobacterium tuberculosis*. The compounds acted as effective-medium potency inhibitors, with K_I_s in the range of 54.6 nM–1.8 µM against hCA I, in the range of 32.1 nM–5.5 µM against hCA II and of 127 nM–2.12 µM against mtCA 3.
